# Supplementary material for: Citizen Stance towards Mandatory COVID-19 Vaccination and Vaccine Booster Doses: A Study in Colombia, El Salvador and Spain
Source: Vaccines (Basel). 2022 May 15;10(5):781. doi: 10.3390/vaccines10050781 (PMC9147164; doi:10.3390/vaccines10050781)
Supplement: Supplementary file 1 [file vaccines-10-00781-s001.zip › vaccines-1712712-supplementary.pdf]

SUPPLEMENTARY MATERIAL

The following online survey (in Spanish) was administered in El Salvador (and the same version but adapted to Spain and Colombia):

El cuestionario recoge las siguientes preguntas con el siguiente encabezado:

El presente estudio tiene como objetivo examinar el impacto que tienen distintos factores sociodemográficos así como aquellos relacionados propiamente con la COVID-19 (como la posible sintomatología experimentada tras la vacunación, comorbilidades, o información recibida entre otros) en cuatro tipos de actitudes negativas frente a la vacuna: 1) desconfianza sobre el beneficio de la vacuna, 2) preocupación sobre los efectos de un futuro imprevisible 3) preocupaciones sobre la especulación comercial, y 4) preferencia para la inmunidad natural. Usted debe saber que el presente estudio es totalmente anónimo. También nos gustaría conocer su actitud hacia una posible vacunación obligatoria y sobre su intención de vacunarse con una posible tercera o consecutivas dosis. Este estudio ha sido aprobado en cada uno de los Comités de Ética del país participante. Si desea participar pulse aquí y si no lo desea esto es todo. Al igual que ocurre cuando usted realiza una búsqueda en Google o utiliza sus servicios, esta empresa sí podría identificarle y recopilar sus datos. Por ello, deberá aceptar la política de privacidad de Google pulsando en el siguiente enlace (link) si desea contestar al siguiente cuestionario.

Le pedimos que sus respuestas se ajusten lo más posible a la realidad.

¿Te has vacunado contra la COVID-19?

- No, porque no he querido
- No, porque tuve la COVID-19
- No, porque aún no me lo han propuesto
- Sí, pero solo parcialmente me han puesto una dosis y son necesarias dos dosis
- Solo parcialmente porque tuve la COVID-19
- Sí, y me han puesto las dosis necesarias (ya sea porque solo necesita una dosis como la de Janssen o te han puesto las dos dosis como la de Pfizer, Sinovac o AstraZeneca)

Edad

- <18 años
- 18-25 años
- 26-35 años
- 36-50 años
- 51-64 años
- >65 años

Sexo

- Mujer
- Hombre

¿Te has vacunado contra la COVID-19?

- No, porque todavía no me han dado prioridad
- No, porque tenía el COVID-19
- No, porque me negué
- Sólo parcialmente (una dosis)
- Sólo parcialmente porque tenía el COVID-19
- Sí, y recibí las dosis necesarias

A continuación, te pedimos que te centres en las vacunas para la COVID-19. Las respuestas se calificarán con una puntuación de seis puntos en una escala donde 1 "totalmente de acuerdo" a 6 "totalmente en desacuerdo".

- Me siento seguro después de ser vacunado.
- Puedo confiar en las vacunas para detener enfermedades infecciosas graves.
- Me siento protegido después de vacunarme.

- Aunque la mayoría de las vacunas parecen seguras, puede haber problemas que aún no hemos descubierto.
- Las vacunas pueden causar problemas imprevistos en los niños.
- Me preocupan los efectos desconocidos de las vacunas en el futuro.
- Las vacunas generan mucho dinero para las compañías farmacéuticas, pero no hacen mucho para la gente común.
- Las autoridades promueven la vacunación con fines económicos, no para la salud de las personas.
- Los programas de vacunación son una gran estafa.
- La inmunidad natural dura más que una vacuna.
- La exposición natural a virus y gérmenes brinda la protección más segura.
- Naturalmente, estar expuesto a enfermedades es más seguro para el sistema inmunológico que estar expuesto a través de la vacunación.

¿Qué vacuna te han administrado?

- AstraZeneca
- Pfizer
- Moderna
- Janssen
- Sinovac
- Otra…

¿Cuándo te vacunaste (de la última dosis)?

- Hace menos de 3 meses
- De 3 a 6 meses
- De 6 a 12 meses

- Más de 12 meses

¿Has tenido algún efecto secundario derivado de la vacuna en la PRIMERA dosis?

- No
- Fiebre <38 grados
- Fiebre de 38 o más grados
- Mialgias (dolores musculares)
- Dolor, enrojecimiento o hinchazón en la zona del pinchazo
- Náuseas
- Vómitos
- Rash cutáneo (erupción cutánea) o prurito (picor de la piel)
- Dolor de cabeza o sensación de pesadez
- Diarreas
- Somnolencia o sensación de mareo
- Disminución del apetito
- Sudoración excesiva
- Escalofríos
- Ganglios linfáticos agrandados
- Otra…

¿Has tenido algún efecto secundario derivado de la vacuna en la SEGUNDA dosis?

- No
- Fiebre <38 grados
- Fiebre de 38 o más grados

- Mialgias (dolores musculares)
- Dolor, enrojecimiento o hinchazón en la zona del pinchazo
- Náuseas
- Vómitos
- Rash cutáneo (erupción cutánea) o prurito (picor de la piel)
- Dolor de cabeza o sensación de pesadez
- Diarreas
- Somnolencia o sensación de mareo
- Disminución del apetito
- Sudoración excesiva
- Escalofríos
- Ganglios linfáticos agrandados
- Otra…

Si tuviste algún efecto secundario, ¿en qué dosis fueron más fuertes?

- Tuve efectos secundarios en la primera dosis, pero solo me he puesto una dosis
- En las dos dosis
- En la primera dosis tuve más efecto que en la segunda dosis
- En la segunda dosis tuve más efecto que en la primera dosis
- Otra…

Si tuviste algún síntoma o sigues con algún síntoma derivado de la vacuna, ¿cuánto tiempo te duró o te está durando?

- <12 horas

- 12-24 horas
- 24-48 horas
- >48 horas
- Otra…

¿Cuánto tiempo tardó en aparecer el primer síntoma derivado de la vacuna?

- <5 horas
- 5-24 horas
- Más de 1 día
- Después de una semana o más

¿Tomaste alguna medicación para mitigar esos posibles síntomas?

- No, no tomé nada
- Sí, paracetamol
- Sí, ibuprofeno
- Sí, paracetamol e ibuprofeno
- Otra…

Si tomaste alguna medicación, ¿cuándo la tomaste?

- Inmediatamente antes de ponerme la vacuna y después también
- Después de la vacuna cuando me empecé a encontrar mal
- Me tomé medicación (antes o después) aunque no me encontré mal en ningún momento
- Otra…

¿Has pasado o crees que has pasado la Covid-19?

- Sí, lo he pasado y tuve PCR positiva

- Sí, lo he pasado y tuve prueba positiva que lo demostró (distinta a la PCR, p.ej. serología positiva)
- Sí, yo creo que la he pasado, aunque no me he hecho ninguna prueba o la prueba me salió negativa
- No, no la he pasado

Sí has contestado que has pasado o crees haber pasado la Covid-19, ¿hace cuánto la pasaste?

- Hace menos de 3 meses
- Entre 3 y 6 meses
- Hace más de 6 meses

¿Si pasaste la Covid-19 podrías indicar los síntomas que tuviste?

- Fiebre
- Tos seca
- Cansancio
- Molestias y dolores
- Dolor de garganta
- Diarrea
- Conjuntivitis
- Dolor de cabeza
- Pérdida del sentido del olfato o del gusto
- Erupciones cutáneas o pérdida del color en los dedos de las manos o de los pies
- Dificultad para respirar o sensación de falta de aire
- Dolor o presión en el pecho
- Incapacidad para hablar o moverse

- Otros

Escribe tu peso (en kg) y talla (en cm). Por favor intenta ser lo más preciso posible para sacar los resultados más certeros que ayuden a la investigación

Por favor indica tu grupo sanguíneo y Rh si lo conoces

- A
- B
- AB
- 0

RH

- Positivo
- Negativo

Indique por favor si tiene algún problema de salud de relevancia:

- Hipertensión
- Diabetes Mellitus tipo 2
- Enfermedades autoinmunes importantes (Lupus, Síndrome de Sjögren, Diabetes tipo 1, Dermatomiositis…)
- Insuficiencia Renal Crónica
- Enfermedades respiratorias (EPOC, asma…)
- Cáncer
- Otras

¿Te informaron previamente de los síntomas que podrías tener antes de vacunarte y de la posibilidad de paliarlos con determinadas prácticas (p.ej. paracetamol, AINES, frío local…)?

- Sí, de forma detallada

- Sí, pero vagamente
- No

¿En caso de haberte vacunado con dos dosis, cuánto tiempo pasó entre cada dosis?

- 21 días
- 28 días
- 3 meses
- Otros

Si te has vacunado ya y tuvieras la oportunidad de volver atrás, ¿te volverías a vacunar?

- Sí claro
- Sí, pero me lo pensaría más
- Tendría dudas
- No, no me compensa por los síntomas que he tenido

¿Qué tipo de estudios tienes (finalizados)?

- Relacionados con Ciencias de la Salud y universitarios (Medicina, Enfermería…)
- Universitarios pero no relacionados con Ciencias de la Salud
- No Universitarios

¿Has nacido en El Salvador?

- Sí
- No

Indica por favor en qué sector estás:

- Sanitario
- No sanitario

Por favor indique su profesión si estás en el sector sanitario:

- Estudiante
- Desempleado
- Jubilado
- Médico/a
- Enfermero/a
- Odontólogo/a
- Fisioterapeuta
- Terapeuta ocupacional
- Nutricionista
- Psicólogo/a
- Auxiliar (de enfermería, laboratorio, rayos…)
- Personal administrativo
- Comercial
- Otros (especificar)

¿Consideras que has estado en la primera línea de lucha contra la COVID-19?

- Sí
- No
- En algunas ocasiones

Consideras la vacunación OBLIGATORIA contra la COVID-19 una violación de los derechos humanos (por ejemplo, la libertad individual)?

- Sí

- No

¿Crees necesario que la vacunación contra la COVID-19 se haga obligatoria?

- Sí, para todo el mundo
- Sí, pero solo para determinados sectores (p.ej. auxiliares en residencias para ancianos, todos los profesionales sanitarios...). Si ha marcado en la anterior respuesta "Sí, pero solo para determinados sectores" por favor indique para que sector
- No
- Otra (especificar)

¿Qué medidas consideras más apropiadas para hacer que la gente se vacune contra la COVID-19?

- Pedir una prueba PCR o antígenos negativo para hacer cualquier actividad (ir al cine, al teatro, de restaurantes, viajar...)
- Suspender de empleo y/o sueldo
- No considero que se deba tomar ninguna medida
- No permitir el ingreso a establecimientos como centros comerciales, oficinas, instituciones de educación, etc
- Otras. Si ha marcado en la anterior respuesta "otras" por favor indica cuáles.

¿Cómo considera se podría mejorar la información y comunicación a la población salvadoreña acerca de la COVID-19?

- Anuncios de televisión, radio, periódicos…
- Folletos informativos otorgados en el centro de salud u hospital
- Personal sanitario que explique con mayor detalle a la población la vacuna y sus efectos
- Cursos
- Otros (especificar)

Si has recibido las dosis necesarias ¿te vas a vacunar de la tercera dosis o de dosis posibles consecutivas?

- Sí, claro
- Tendría dudas
- No

Después de haberte vacunado has contraído la COVID-19

- No
- Sí, pero solo me había puesto una dosis y eran necesarias dos (p.ej. en Pfizer, Sinovac, Moderna o Astrazeneca/vaxzevria)
- Sí, y me había puesto las dosis necesarias (p.ej. una en el caso de Janssen o dos en el caso de Pfizer, Sinovac, Moderna o Astrazeneca/vaxzevria)
- Otra (especificar)

Por último, por favor céntrate en las vacunas para la COVID-19 y responde en una escala de 1 "totalmente de acuerdo" a 6 "totalmente en desacuerdo" con lo que más te identificas.

- Me siento seguro después de ser vacunado.
- Puedo confiar en las vacunas para detener enfermedades infecciosas graves.
- Me siento protegido después de vacunarme.
- Aunque la mayoría de las vacunas parecen seguras, puede haber problemas que aún no hemos descubierto.
- Las vacunas pueden causar problemas imprevistos en los niños.
- Me preocupan los efectos desconocidos de las vacunas en el futuro.
- Las vacunas generan mucho dinero para las compañías farmacéuticas, pero no hacen mucho para la gente común.
- Las autoridades promueven la vacunación por fines económicos, no para promover la salud de las personas.

- Los programas de vacunación son una gran estafa.

- La inmunidad natural dura más que la que genera una vacuna.

- La exposición natural a virus y gérmenes brinda la protección más segura.

- Naturalmente, estar expuesto a enfermedades es más seguro para el sistema inmunológico que estar expuesto a través de la vacunación.

ESO ES TODO, MUCHAS GRACIAS POR CONTESTAR Y CONTRIBUIR AL AVANCE DE LA CIENCIA
